# A Pooled Data Analysis to Determine the Relationship between Selected Metals and Arsenic Bioavailability in Soil

**DOI:** 10.3390/ijerph15050888

**Published:** 2018-04-30

**Authors:** Kaihong Yan, Ravi Naidu, Yanju Liu, Ayanka Wijayawardena, Luchun Duan, Zhaomin Dong

**Affiliations:** 1 Global Centre for Environmental Remediation, the Faculty of Science and Information Technology, University of Newcastle, University Drive, Callaghan, NSW 2308, Australia; kaihong.yan@uon.edu.au (K.Y.); ravi.naidu@newcastle.edu.au (R.N.); yanju.liu@newcastle.edu.au (Y.L.); Ayanka.Wijayawardena@newcastle.edu.au (A.W.); luchun.duan@newcastle.edu.au (L.D.); 2 Cooperative Research Centre for Contamination Assessment and Remediation of the Environment (CRC CARE), Callaghan, NSW 2308, Australia

**Keywords:** arsenic, bioavailability, soil properties, pooled study

## Abstract

Chronic exposure to arsenic (As) is a global concern due to worldwide exposure and adverse effects, and the importance of incorporating bioavailability in the exposure assessment and risk assessment of As is increasing acknowledged. The bioavailability of As is impacted by a number of soil properties, such as pH, clay and metal concentrations. By retrieving 485 data from 32 publications, the aim of this study was to determine the relationship between selected metals (Fe and Al) and As bioavailability. In present study, the bioaccessibility (BAC) data measured by in vitro approaches were converted into bioavailability data based on the previously determined relationship between BAC and bioavailability. The As relative bioavailability (RBA) was summarized to be 24.36 ± 18.49%, which is in the range previously reported. A significant association between Fe concentration and the bioavailability of As was observed while this association varied for different types of RBA data. This disparity may suggest a non-reliable association between Fe and As bioavailability. The correlations between logarithmically transformed total content of Fe + Al and As bioavailability is then outlined: RBA = (−8.40 ± 1.02) × Ln(Fe + Al) + (58.25 ± 4.09), R^2^ = 0.25, *p* < 0.001, *n* = 212. Jackknife resampling was also applied to validate the relation between total content of (Fe + Al) and As bioavailability, which suggested that the relation is robust. This is the first pooled study to address the relations between selected metal concentrations and As bioavailability, which may provide some implications to establish soil properties-based RBA prediction for As.

## 1. Introduction

Chronic exposure to arsenic (As) is a global concern due to worldwide exposure and adverse effects. Both epidemiological and animal studies have convincingly identified that As exposure can result in both cancer and non-cancer risks [1,2]. It is necessary to reduce as exposure from all pathways to eliminate As risk. Due to rapid urbanization all over the world, especially in Asia, exposure to soils with high As levels is becoming a threat [3]. The importance of incorporating soil bioavailability into the exposure assessment and health risk assessment of pollutants is growingly acknowledged for realistic risk assessment and remediation strategies [4].

Initially, in vivo studies using animal models (such as swine, rats, mice, and rabbits) were proposed to estimate As RBA [5]. The in vitro method (BAC) has been suggested to act as a surrogate measure of As RBA if a validated IVIVR can be achieved, considering in vivo methods are always time-consuming and costly [6]. Several IVIVRs were produced previously for As. For example, a number of researchers have reported the correlation between As RBA and As BAC using diverse in vitro assays, including in vitro gastrointestinal extraction method (IVG), physiologically- based extraction test (PBET), the Unified Bioaccessibility Research Group of Europe Method (UBM) and the Solubility Bioaccessibility Research Consortium assay (SBRC) [5,7].

Although the in vitro approaches promise to provide a rapid and robust prediction of RBA, the factors influencing the bioavailable As in soil are not fully understood. Generally, As in a soil can be grouped into several fractions including: As-precipitates, As sorbed to clays, hydrous oxides and organic matter, and As within the matrix of soil minerals, the soluble As in the soil solution. These different fractions are all in dynamic equilibrium with each other [8], and the only soil fraction directly available for biological systems is the As within soil solution [9]. Hence the factors that influence the concentration and speciation of As in soil solution will significantly affect the bioavailability of As to biota. Soil properties which have an effect on As bioavailability include concentrations of metals or metalloids, pH, clay and hydrous oxide content, organic matter and redox conditions [10]. In particular, a number of studies have reported the influences from some other metals present in the soil [11]. For instance, Bradham et al. [11] observed that 80% and 62% of the variability in estimates of As RBA and BAC can be attributed to the log-transformed sum content of iron (Fe) and aluminium (Al) in the soil. Similarly, As sorption on ferrihydrite [${\mathrm{Fe}}_{5}^{3+}O_{3}{\left(\mathrm{OH}\right)}_{9}$] and corundum (Al_2_O_3_) were reported by Beak et al. [12,13]. The results demonstrated that the BAC of As(V) adsorbed to ferrihydrite is related to the As(V) concentration, the As(V) adsorption maximum, the arsenate binding capacity of soil. As a redox-sensitive element, As can sorb strongly to minerals such as (Mn) oxides and Fe oxides under oxic conditions [14]. Takahashi, et al. [15] has demonstrated that the iron redox chemistry is the predominating factor regulating As mobility, and Ackermann, et al. [16] also found a close correlation of As with Mn and Fe at the reference site through sequential extraction.

Knowledge of the behavior of bioavailable As associated with metals content in soil will provide an understanding of the exposure of As-contaminated soil to humans. Although the impact of major metal concentrations on the bioavailability of As were acknowledged, the predominating factors and the relation varied amongst the literature [11]. Related on this topic, there is still no general consensus. The statistical relations between the metal concentrations and As bioavailability are not transparent or not systematically carried out. A pooled study may reduce such uncertainties [17]. In this study, the bioavailability and BAC data, soil properties data and metal concentrations were retrieved to address how these descriptors impact the bioavailability of As. Based on such analysis, this study may provide some implications to establish soil properties-based RBA predictions for As.

## 2. Methods and Materials

### 2.1. Data Collection

The first step for establishing the relationship between metal concentrations and As RBA is data collection. The related data in this study includes metal concentrations, BAC data and RBA data. As, Fe, Al were commonly used in previous studies, and were selected to represent the metals in this study. Meanwhile, some studies suggested that phosphorus (P) and pH are important factors in controlling As bioavailability in soils, and thus the data related on P and pH were also collected.

As mentioned, both in vivo and in vitro approaches were commonly used to measure RBA in the previous literature, and therefore the data involved in the two methodologies were both used. The BAC data were converted to RBA data by using the IVIVR well summarized in Table 1 [5,18,19], which were defined as in vitro RBA in this study.

To retrieve the data, firstly an extensive literature search (for analyses published between 1950 and 2018) using ‘Arsenic exposure’ & ‘Soil’ & ‘RBA or BAC’ as the keywords was done and checked by two co-authors (databases included Pubmed, Web of Science, Medline). More than 1000 titles/abstracts were retrieved and each study was individually checked for its relevance with respect to As bioavailability, metal and soil properties. The basis of selecting studies as follows: (a) published in an international peer-reviewed journal; (b) for the repeated data in two or more publications, the study providing the most complete information was chosen; and (c) the data of RBA, metal and soil properties are available for individual soil samples.

Thus, 183 publications were identified initially. After that, we carefully checked the full text to confirm the data eligibility. Finally, 32 publications with 485 individual data were selected, as summarized in Supplementary Material Table S1.

### 2.2. Model Development

To establish the relationship between metals and the bioavailability of As, different combinations of single variable were proposed: concentration for As, Fe, Al, P, (Fe + Al), (Fe + Al)/As, (Fe + Al)/(As + P). Considering the metal concentration was usually log-normal distributed, the log-transform algorithm was applied for these variables. Meanwhile, for each regression, the impact from pH was included. Also, since both RBA and BAC data were adopted, the models were carried out for in vivo RBA data, in vitro RBA data and all combined data, respectively. The outliers were excluded before conducting the regression. A Jackknife resampling was adopted to evaluate model robustness: data from each study was removed alternatively, and the regression was re-conducted to obtain the model parameters. All the models were performed by Matlab 2016b (MathWorks, Natick, Massachusetts, United States ); and all statistical analysis were done by IBM SPSS 19 (IBM, Armonk, New York, United States).

## 3. Results

### 3.1. Data Description 

Table 2 summarizes selected characteristics of the test soils. For the data extracted from the 32 publications, total As concentration ranged from 4.4 to 310,000 mg/kg, with a mean of 3180 ± 18,841 mg/kg. The high coefficient of variation (CV) is attributed to the As concentrations being subjected to log-normal distribution (S-W test, *p* < 0.1). The Fe content in the soil is the highest (53.14 ± 63.46 g/kg), followed by the Al (23.79 ± 21.58 g/kg) and P (1.06 ± 1.40 g/kg). Soil pH ranged from 2.1 to 9.

The RBA measurements, which ranged from 1.99 to 90.67% with a low mean value of 23.04% ± 17.71% (*n* = 254), varied considerably amongst all literature. Although this value is 17.72% lower than that RBA (27.80% ± 18.51%, *n* = 218) based on BAC prediction (Table 1), no significant differences were observed between the two types of RBA. This comparison may indicate that RBA data based on BAC translation is reliable. By combing the two types of RBA, the anticipated value of the RBA was 24.36% ± 18.49%, which is similar to the value reported by a previously conducted meta-study [20].

### 3.2. Model Development 

The correlation between pH and RBA was tested firstly. The results showed a poor R^2^ of 0.0016, which may illustrate that pH did not directly impact As RBA. Thus, this factor was excluded when regressing other descriptors and RBA. We summarized the regression results after applying log-transform algorithm to descriptors tabulated in Table 3. 

**Figure 1:**
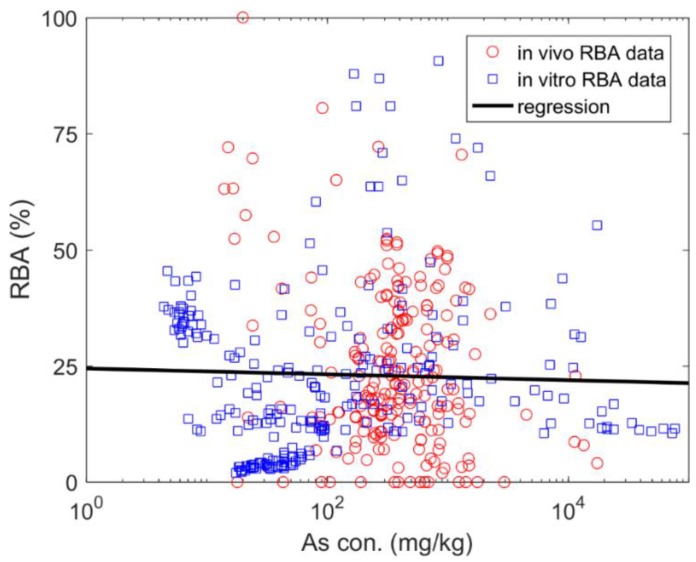
The relation between arsenic (As) concentration (con.) and its relative bioavailability (RBA).

As shown, a significant negative correlation was demonstrated between RBA and total As concentration (*p* < 0.001, *n* = 220) with a poor R^2^ (0.07), while no correlation can be issued for RBA data based on BAC prediction (*p* = 0.71). Such a difference resulted in a non-significant correlation between the combined RBA and total As concentration as plotted in Figure 1.

**Figure 2:**
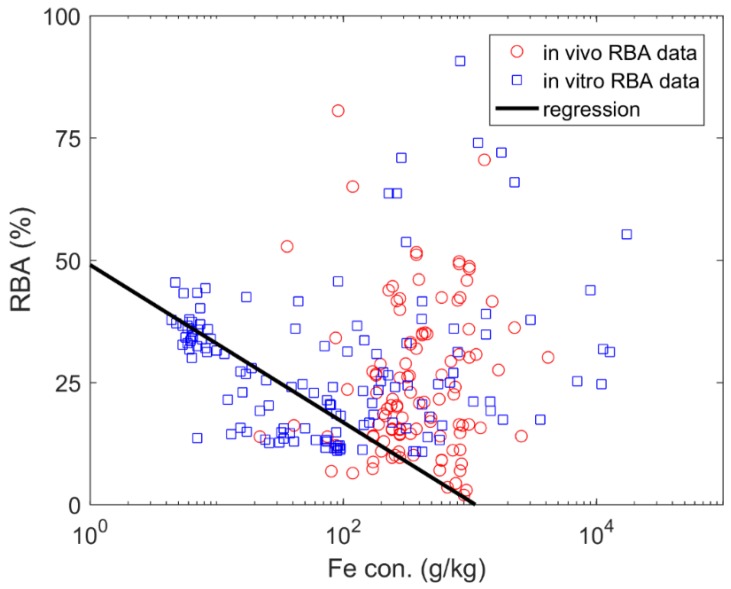
The relation between iron (Fe) concentration (con.) and arsenic relative bioavailability (RBA)**.**

Linear regression models using Fe were able to predict in vivo RBA and in vitro RBA with R^2^ values of 0.27 and 0.11, respectively (Table 3 and Figure 2). Further statistical analysis of the residuals indicate that the model developed is highly significant, which is supported by a small residual in relation to the regression model as illustrated by the Durbin-Watson (D-W) factor of 1.63. A positive autocorrelation of the random error is suggested when the Durbin-Watson values near 0, and a value of 2 indicates no correlation.

Although some studies indicated that As bioavailability can be impacted by Al and P, such a significant relation was not observed in this study. As Tabulated in Table 3, the content of Al and P was poorly related to the RBA, with R^2^ of 0.037 and 0.02 for the combined RBA data, respectively.

**Figure 3:**
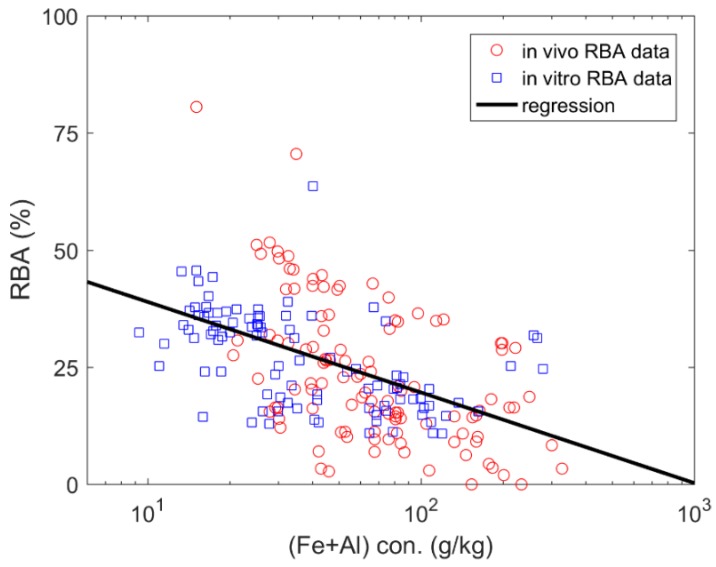
The relation between total content of the iron (Fe) plus aluminium (Al) concentration (con.) and arsenic relative bioavailability (RBA).

An enhanced performance was illustrated when using total content of Fe and Al as the predictors in the linear regression models (Figure 3). As shown in Table 3, the R^2^ were estimated to be 0.29, 0.27 and 0.25 for in vivo RBA data, in vitro RBA data and combined data, respectively. 

Meanwhile, the parameters presented in the three models were quite similar. For example, the slopes were calculated to be −11.58, −6.58 and −8.40 for in vivo RBA data, in vitro RBA data and combined data, respectively. For data combined, the median and mean of absolute prediction error is approximate 35% and 59%, respectively. The soil characteristics data used in this prediction is available upon request.

Regression analysis identified that using the ratio between total Al and Fe concentrations and As concentrations (ID 6 in Table 3) were able to significantly explain about 20% RBA variation. However, the results varied between various RBA data. As exemplified, the slopes ranged from 2.42 to 29.60% and the intercepts were calculated to 17.67% for in vitro RBA data and 62.47% for in vivo RBA data. This high heterogeneity suggested this descriptor may not be robust. Similar result was obtained when using the ratio between total Al and Fe concentrations and total As and P concentrations (ID 7 in Table 3) to predict RBA data. Although significant regressions were obtained for both in vivo RBA and in vitro RBA data, a significant regression was not anticipated for combined data. This disparity may indicate this integrated descriptor may be not suitable to predict RBA.

### 3.3. Model Validation 

In this study, the model using the log-transform of the total content of Fe and Al to predict the RBA is preferred due to the high significance and little variation amongst various RBA data. To further validate this model, we also attempted to use an exponential function to predict the RBA:

(1) $$RBA=-0.089\times exp^{Ln\left(Fe+Al/As\right)}+31.52$$

The R^2^ for Equation (1) was calculated to be 0.17 (*p* < 0.001, *n* = 212), which was about 30% lower than that based on linear regression. When utilizing the total Fe and Al content as the descriptor to predict RBA, 212 data from 11 publications were highlighted. To test the impact from each study on model performance, jackknife resampling was employed. All the regressions were significant with R^2^ ranged from 0.17 to 0.34. Most slopes varied with a narrow interval, from −9.01 to −7.64, which may suggest no influential study existed in the regression. However, when deleting data from Juhasz, et al. [21], the slopes were −10.66, which was lower than all other models. A possible reason is the sample size in Juhasz et al.'s paper [21] was 32, which was the largest. 

## 4. Discussion

Although primates are the first option for bioavailability studies due to their close relationship to humans, only a few studies have been carried based on this animal model due to prohibitive costs. Commonly, scientists used swine and rodent models to measure RBA [22]. Of the 32 publications collected, 15 publications employed in vivo approaches and the other 17 reports utilized in vitro approaches. For the 15 publications related to in vivo protocols, RBA data from seven publications were based on swine models, and six publications adopted rodents to measure RBA.

Brattin and Casteel [23] described a method for measuring the RBA of As in soil and other soil-like media using young swine, on the basis of urinary As excretion as the measurement endpoint. The authors stated the results of this investigation indicate that swine is a useful model for quantifying GI absorption of As from different test materials. By reviewing data on RBA of As in soil, U.S. EPA [24] concluded that different animal models may yield similar results: in details, when assessing four soils from the orchard, the results from swine, monkey and mouse were not significantly different. Similarly, when comparing three standard reference material assays, the differences for RBA measurements between swine and mouse were also not observed. However, when determining the RBA for the nine test soils, it seems there is a tendency that the swine assay yielded higher RBA estimates than the rodent assay. Nevertheless, it is challenge to use such limited results to draw a consistent conclusion.

As mentioned, the RBA data from 17 publications was from in vitro approaches, including the SBRC, UBM, IVG and PBET. Some previous studies have compared the BAC results from various in vitro assays. For example, Oomen, et al. [25] employed five in vitro methods (SBET, DIN, RIVM, SHIME, TIM) to assess the BAC for two historically contaminated soils and one NIST reference soil. The BAC values amongst the five in vitro methods varied considerably. Although the parameters for each in vitro method are different, the disparity in As BAC values was due to the differences in the gastric pH of each method as suggested by Oomen et al. [25]. Rodriguez, et al. [26] compared as BAC based on IVG and PBET methods for mining and smelting material, and the results showed that the PBET method produced relatively lower As BAC values than those measured by the IVG method. The authors indicated that the possible reason is the different amount of pepsin used in the two in vitro solutions, which was able to elevate the levels of hydrolyzed products in order to enhance the As solubilization [26]. Further investigations are still required to evaluate the performance of in vitro assays though some preliminary information was available relating the variability in As BAC amongst various in vitro approaches.

In this study, only the BAC data from gastric phase rather than intestine phase was used, in order to reduce the heterogeneity between different phases. For the SBRC and IVG methods, As BAC based on gastric-phase values were usually higher than that based on intestinal phase [18]. With increase in pH from gastric- to intestinal-phase, dissolved Fe from gastric-phase dissolution becomes oversaturated, and hydrolyzed Fe species precipitate as amorphous Fe structures and thereby it reduced the concentration of dissolved as in the intestinal phase.

Therefore, considering the uncertainties amongst in vitro assays and in vivo models, a IVIVR is mandatory prerequisites for scientific acceptance before these in vitro assays to be used as a surrogate measurement. In present study, the Equations reviewed by [5,18,19] were utilized. Actually, a number of studies have previously investigated the IVIVRs. For SBRC methods, Bradham et al. [11] proposed the following equations to bridge the SBRC BAC and RBA based on 11 mining and smelter soils:

(2) RBA=0.72×BAC+5.64

Comparing to the Equations used in this study, the slope in Equation (2) was relatively lower, while the intercept was relatively higher. The slopes and intercepts from other studies ranged from 0.69 to 1.67 and 1.89 to 6.85, which may suggest high uncertainties for the existing IVIVRs. Similar phenomenon was also observed for other in vitro approaches, and a meta-analysis may help reduce such uncertainties [27]. Further validation and dissemination of As in IVIVRs is beneficial for BAC data to be appropriately used to determine the RBA of As in soils.

It is not surprising that the total content of Fe plus Al can reduce As bioavailability by affecting solubility of metals in soils. As stated by Yang, et al. [28], the logarithmically transformed Fe content explained 56% of the variability in the relative adsorption of the soils, comparing to all of the variables together that explained only 66% of the variability. Bradham et al. [11] also indicated that sorption of As to Fe and Al oxides reduces As solubilization and thereby reduces As RBA and BAC. However, this is the first pooled study to address the relations between these metals and As bioavailability.

Juhasz, et al. [29] identified that the total As plus Fe concentrations were the variables that can best describe the variation in in vivo bioavailability of As. However, when entering the As concentrations as the descriptor in the model 5 (Table 3), the R^2^ was calculated to be 0.26, which approximately equal to the result of the model 5 with origin descriptor. This comparison indicated adding the As concentration as a new independent variable hardly improves the regression model. Thus, the descriptor of As concentration was excluded in model 5.

Commonly, a significant relation between RBA or BAC of As and pH was reported by numerous studies, as pH is critical to control As sorption in soils [30]. By controlling metal dissolution and precipitation, soil pH is able to modify the metal solubility and availability. In particular, soil pH regulates the ionization of pH-dependent ion-exchange sites on organic matter and metal oxide clay minerals to influence the availability of cationic metals. Also, some studies have reported the integration of pH and Fe content controls the As sorption in soils [30]. The Fe oxides can alter surface charges in response to soil solution pH [29], and the negative surface charge on Fe oxides also increases with the pH increase. Generally, a low soil pH favours the adsorption of As onto colloidal material such as Fe oxides due to their high positive surface charge (and large surface area) under acidic conditions [29]. A rise in pH increases the negative potential on the plane of adsorption of Fe oxide surfaces as well as changing the nature of the charged As species in solution [29]. However, in this pooled study, the impact from pH was not exhibited, which may be due to the high literature heterogeneities.

The effect from phosphate was not determined in this study either, although some authors stated that the phosphate can decrease the intestinal absorption of As [31]. Potentially, the high P/As molar ratio may reduce As RBA due to P competition of the in vivo system. The effects from some other metals (such as Mn and Mg) were not determined since most literature did not report the concentrations of these two elements, while Juhasz et al. [31] demonstrated the efficacy of Mn and Mg surfaces to sorb As. In detail, due to the elevated concentrations of both Mn and Mg, dissolution and precipitation processes may decrease the as absorption and availability in gastrointestinal tract.

This study only addressed the impact from selected metals on As bioavailability. The effects of individual soil properties on As bioavailability is difficult to determine, considering the soil properties are always intercorrelated. For example, cation-exchange or ligand-exchange may account for the linkage between clay minerals and metals that occur on iron and aluminum oxides clay. For this reason, soil clay content typically is highly correlated with Fe, Al content and cation exchange capacity [32]. Metals are bound strongly to soils that are rich in clay or organic matter. Considering many soil parameters have more than one binding mechanism and are strongly intercorrelated with other soil parameters, the casual effects are hard to distinguish. 

## 5. Conclusions

Based on an extensive literature search, the RBA was summarized to be 24.36% ± 18.49%, which was in the reported range of previous literature. No significant relations between single element concentration (such as As, Fe, Al and P) and bioavailability of As was observed. This study confirmed that the logarithmically transformed total content of Fe + Al can explain approximate 25% variation of As bioavailability, and model validation based on jackknife resampling suggested this model is robust.

## Figures and Tables

**Table 1:** Summarized *in vitro-in vivo* relations for arsenic [5,18,19].

Methods	RBA Measurement	Equations (% for RBA and BAC)
SBRC-G	AUC/blood	RBA = 0.99 × BAC + 1.89, R^2^ = 0.92
IVG-G	AUC/blood	RBA = 0.89 × BAC + 5.14, R^2^ = 0.69
PBET-G	AUC/blood	RBA = 0.60 × BAC + 10.20, R^2^ = 0.59
UBM-G	AUC/blood	RBA = 0.99 × BAC + 0.80, R^2^ = 0.52

RBA: relative bioavailability; BAC: Bioavailability and bioaccessibility; AUC: the area under the curve.

**Table 2:** Summarized Information for Model Descriptors.

Independent Variables	Range	Mean ± Std	Median	Study Number	Data Number
As (mg/kg)	4.4–310,000	3180 ± 18,841	268	30	462
Fe (g/kg)	2.09–317	53.14 ± 63.46	29.96	17	261
Al (g/kg)	0.67–98.9	23.79 ± 21.58	15.15	11	214
P (mg/kg)	4–8159	1057 ± 1401	550	8	154
pH	2.1–9	6.75 ± 1.55	7	18	280
(Fe + Al) (g/kg)	9.3–328.7	79.13 ± 72.61	51.44	11	212
(Fe + Al)/As	12.15–4807	754.99 ± 1134	225.88	10	197
(Fe + Al)/(As + P)	5.27–259.7	59.44 ± 55.6	37.50	6	145

**Table 3:** Regressions for relation between selected metals and arsenic bioavailability.

ID	Independent Variables	Data Type	Equations
1	Ln(As) (mg/kg)	RBA (in vivo)	Y = (−4.12 ± 1.02) × X + (48.85 ± 6.12), R^2^ = 0.07, *p* < 0.001, *n* = 220;
		RBA (in vitro) *	Y = (0.18 ± 0.47) × X + (20.06 ± 2.45), R^2^ = 0.0006, *p* = 0.71, *n* = 242;
		Combined	Y = (−0.27 ± 0.42) × X + (24.43 ± 2.37), R^2^ = 0.0009, *p* = 0.52, *n* = 462;
2	Ln(Fe) (g/kg)	RBA (in vivo)	Y = (−10.35 ± 1.53) × X + (62.2 ± 5.86), R^2^ = 0.27, *p* < 0.001, *n* = 126;
		RBA (in vitro) *	Y = (−4.81 ± 1.22) × X + (41.22 ± 3.85), R^2^ = 0.11, *p* < 0.001, *n* = 135;
		Combined	Y = (−6.99 ± 1.02) × X + (49.02 ± 3.18), R^2^ = 0.19, *p* < 0.001, *n* = 261;
3	Ln(Al) (g/kg)	RBA (in vivo)	Y = (−0.26 ± 1.74) × X + (24.58 ± 5.01), R^2^ = 0.0002, *p* = 0.88, *n* = 114;
		RBA (in vitro) *	Y = (−7.07 ± 1.11) × X + (45.95 ± 3.16), R^2^ = 0.30, *p* < 0.001, *n* = 100;
		Combined	Y = (−3.17 ± 1.33) × X + (33.99 ± 8.22), R^2^ = 0.037, *p* < 0.01, *n* = 214;
4	Ln(P) (mg/kg)	RBA (in vivo)	Y = (1.57 ± 1.34) × X + (18.05 ± 8.22), R^2^ = 0.02, *p* = 0.23, *n* = 69;
		RBA (in vitro) *	Y = (1.03 ± 0.91) × X + (21.55 ± 8.46), R^2^ = 0.007, *p* = 0.44, *n* = 85;
		Combined	Y = (1.61 ± 1.12) × X + (18.02 ± 5.68), R^2^ = 0.02, *p* = 0.07, *n* = 154;
5	Ln(Fe + Al) (g/kg)	RBA (in vivo)	Y = (−11.85 ± 1.79) × X + (73.52 ± 7.59), R^2^ = 0.29, *p* < 0.001, *n* = 114;
		RBA (in vitro) *	Y = (−6.58 ± 1.11) × X + (50.60 ± 4.12), R^2^ = 0.27, *p* < 0.001, *n* = 98;
		Combined	Y = (−8.40 ± 1.02) × X + (58.25 ± 4.09), R^2^ = 0.25, *p* < 0.001, *n* = 212;
6	Ln(Fe + Al)/Ln(As)	RBA (in vivo)	Y = (29.60 ± 10.34) × X + (62.47 ± 7.07), R^2^ = 0.24, *p* < 0.001, *n* = 99
		RBA (in vitro) *	Y = (8.70 ± 2.57) × X + (17.67 ± 2.57), R^2^ = 0.13, *p* < 0.001, *n* = 98;
		Combined	Y = (2.42 ± 1.94) × X + (19.95 ± 1.94), R^2^ = 0.06, *p* < 0.001, *n* = 197;
7	Ln(Fe + Al)/Ln(As + P)	RBA (in vivo)	Y = (−74.67 ± 10.64) × X + (69.17 ± 10.64), R^2^ = 0.22, *p* < 0.001, *n* = 65;
		RBA (in vitro) *	Y = (−82.34 ± 6.09) × X + (71.32 ± 6.09), R^2^ = 0.40, *p* < 0.001, *n* = 80;
		Combined	Y = (−20.38 ± 10.06) × X + (59.30 ± 10.06), R^2^ = 0.07, *p* = 0.002, *n* = 145;

Note: * the RBA data was obtained based on BAC by using Equations from Table 1.

## Supplementary Materials

The following are available online at http://www.mdpi.com/1660-4601/15/5/888/s1, Table S1: The raw data for pooled study.
