# Supplementary material for: A Pooled Data Analysis to Determine the Relationship between Selected Metals and Arsenic Bioavailability in Soil
Source: Int J Environ Res Public Health. 2018 Apr 30;15(5):888. doi: 10.3390/ijerph15050888 (PMC5981927; doi:10.3390/ijerph15050888)
Supplement: Supplementary file 1 [file ijerph-15-00888-s001.pdf]

International Journal of Environmental Research and Public Health

# Supplementary Materials: A Pooled Data Analysis to Determine the Relationship between Selected Metals and Arsenic Bioavailability in Soil

**Kaihong Yan [1,2], Ravi Naidu [1,2], Yanju Liu [1,2], Ayanka Wijayawardena [1,2], Luchun Duan [1,2] and Zhaomin Dong [1,2,*]**

[1] Global Centre for Environmental Remediation, the Faculty of Science and Information Technology, University of Newcastle, University Drive, Callaghan, NSW 2308, Australia; kaihong.yan@uon.edu.au (K.Y.); ravi.naidu@newcastle.edu.au (R.N.); yanju.liu@newcastle.edu.au (Y.L.); Ayanka.Wijayawardena@newcastle.edu.au (A.W.); luchun.duan@newcastle.edu.au (L.D.)

[2] Cooperative Research Centre for Contamination Assessment and Remediation of the Environment (CRC CARE), Callaghan, NSW 2308, Australia

* Correspondence: morrow.dong@newcastle.edu.au; Tel.: +61-2-4913-8705

**Table S1. The raw data for pooled analysis.**

| Reference | Study | RBA | BAC | As (mg/kg) | Fe (g/kg) | Al (g/kg) | P (mg/kg) | pH | Index * |
|---|---|---|---|---|---|---|---|---|---|
| (Bradham et al., 2011) | 1 | 49.9 | | 990 | 20.9 | 11.8 | | 6.1 | 1 |
| | 1 | 50.9 | | 829 | 20.5 | 9.4 | | 6.3 | 1 |
| | 1 | 52.8 | | 379 | 18.9 | 9 | | 5 | 1 |
| | 1 | 11.45 | | 837 | 294.4 | 13.2 | | 7.2 | 1 |
| | 1 | 15.9 | | 244 | 46 | 21.7 | | 7.3 | 1 |
| | 1 | 14.4 | | 173 | 63.4 | 20.9 | | 6.6 | 1 |
| | 1 | 15 | | 6899 | 144.5 | 15 | | 5.2 | 1 |
| | 1 | 40.9 | | 280 | 72.3 | 3.9 | | 2.1 | 1 |
| | 1 | 14.8 | | 4495 | 120.1 | 12.3 | | 2.6 | 1 |
| | 1 | 43.9 | | 601 | 29.2 | 17.2 | | 5 | 1 |
| | 1 | 42.9 | | 1513 | 34 | 10 | | 4 | 1 |
| (Juhasz et al., 2015) | 2 | 10.8 | | 275 | 89.6 | 52.4 | 2567 | | 1 |
| | 2 | 12.9 | | 210 | 60.6 | 44.2 | 2350 | | 1 |
| | 2 | 6.8 | | 81 | 60.3 | 26.7 | 1067 | | 1 |
| | 2 | 10.1 | | 358 | 96.2 | 65.1 | 1710 | | 1 |
| | 2 | 10.9 | | 200 | 52.8 | 29 | 2393 | | 1 |
| | 2 | 18.2 | | 215 | 107 | 73.9 | 2340 | | 1 |
| | 2 | 16.4 | | 981 | 141 | 77.8 | 2294 | | 1 |
| | 2 | 15.7 | | 1221 | 96.8 | 66.9 | 3787 | | 1 |
| | 2 | 45.8 | | 949 | 27.1 | 7.1 | 718 | | 1 |
| | 2 | 30.7 | | 1126 | 18.4 | 2.9 | 578 | | 1 |
| | 2 | 27.5 | | 1695 | 18.2 | 2.4 | 375 | | 1 |
| | 2 | 70.5 | | 1306 | 24.6 | 10.4 | 628 | | 1 |
| | 2 | 36.2 | | 2270 | 32.1 | 14 | 838 | | 1 |
| (Juhasz et al., 2009) | 3 | 63.6 | 89 | 267 | 17.6 | 22.2 | 234 | 8.8 | 0 |
| | 3 | 36 | 43 | 42 | 13.7 | 18.3 | 385 | 8.4 | 0 |
| | 3 | 29.4 | 32 | 1114 | 0 | 16.3 | 874 | 7.8 | 0 |
| | 3 | 24 | 23 | 257 | 25.8 | 27.8 | 242 | 6.4 | 0 |
| | 3 | 36 | 43 | 751 | 14.5 | 10.8 | 422 | 8.3 | 0 |
| | 3 | 45.6 | 59 | 91 | 10 | 5.1 | 130 | 7.5 | 0 |
| | 3 | 25.2 | 25 | 713 | 0 | 94.7 | 3144 | 5.7 | 0 |
| | 3 | 63.6 | 89 | 228 | 17.9 | 22.4 | 2941 | 5.2 | 0 |
| | 3 | 31.2 | 35 | 807 | 23.5 | 10.9 | 546 | 7.6 | 0 |
| | 3 | 13.2 | 5 | 577 | 24.6 | 17.6 | 468 | 6.6 | 0 |
| | 3 | 23.4 | 22 | 190 | 21 | 8.3 | 200 | 8.6 | 0 |
| | 3 | 18.6 | 14 | 88 | 21 | 9.6 | 370 | 8.1 | 0 |
| (Denys et al., 2012) | 4 | 3.5 | | 1500 | | | | 6.8 | 1 |
| | 4 | 3.25 | | 25000 | | | | 7.2 | 1 |
| | 4 | 13.5 | | 105 | | | | 7.9 | 1 |
| | 4 | 3 | | 390 | | | | 7.4 | 1 |

| | | | | | | | | | |
|---|---|---|---|---|---|---|---|---|---|
| | 4 | | | 43 | | | | 7.9 | 1 |
| | 4 | 10.5 | | 247 | | | | 7.7 | 1 |
| | 4 | 4.75 | | 214 | | | | 6.9 | 1 |
| | 4 | 7 | | 297 | | | | 6.7 | 1 |
| | 4 | 10.75 | | 283 | | | | 8.1 | 1 |
| | 4 | 57.5 | | 21 | | | | 6.6 | 1 |
| | 4 | 69.67 | | 61 | | | | 7.9 | 1 |
| | 4 | 33.67 | | 24 | | | | 7.2 | 1 |
| | 4 | | | 18 | | | | 6.9 | 1 |
| | 4 | 100 | | 20 | | | | 7.6 | 1 |
| | 4 | 43 | | 190 | | | | 7 | 1 |
| (Rodriguez et al., 1999; Denys et al., 2012) | 5 | 2.7 | | | 297 | 11.9 | | 2.6 | 1 |
| | 5 | 3.3 | | | 317 | 11.7 | | 2.6 | 1 |
| | 5 | 8.3 | | | 285 | 16 | | 3.1 | 1 |
| | 5 | 22.1 | | | 25 | 18 | | 3.1 | 1 |
| | 5 | 30.1 | | | 166 | 30.2 | | 5.7 | 1 |
| | 5 | | | | 209 | 24.3 | | 7.4 | 1 |
| | 5 | | | | 117 | 36.2 | | 7.7 | 1 |
| | 5 | 28.7 | | | 166 | 32 | | 7.1 | 1 |
| | 5 | 30.1 | | | 172 | 26.4 | | 7.4 | 1 |
| | 5 | 16.4 | | | 183 | 27.4 | | 7.4 | 1 |
| | 5 | 6.2 | | | 70.7 | 75.6 | | 3.9 | 1 |
| | 5 | 42.8 | | | 20.1 | 46.4 | | 4.6 | 1 |
| | 5 | 29.1 | | | 204 | 17.3 | | 7.5 | 1 |
| | 5 | 18.7 | | | 225 | 24.8 | | 7.3 | 1 |
| | 5 | 36.5 | | | 61.7 | 35.6 | | 7.6 | 1 |
| (Rodriguez et al., 1999; Denys et al., 2012; Bradham et al., 2013) | 6 | 39.9 | | 280 | 72.3 | 3.9 | | 2.1 | 1 |
| | 6 | 14.5 | | 4495 | 120.1 | 12.3 | | 2.6 | 1 |
| | 6 | 26.7 | | 182 | 24.1 | 20.5 | | 6.8 | 1 |
| | 6 | 48.7 | | 990 | 20.9 | 11.8 | | 6.1 | 1 |
| | 6 | 49.7 | | 829 | 20.5 | 9.4 | | 6.3 | 1 |
| | 6 | 51.6 | | 379 | 18.9 | 9 | | 5 | 1 |
| | 6 | 11.2 | | 837 | 294.4 | 13.2 | | 7.2 | 1 |
| | 6 | 24 | | 769 | 59.2 | 6.3 | | 5.6 | 1 |
| | 6 | 26.3 | | 336 | 25.5 | 27.4 | | 6.6 | 1 |
| | 6 | 35.2 | | 446 | 49.2 | 71.7 | | 6.6 | 1 |
| | 6 | 20.9 | | 437 | 38.6 | 44.2 | | 6.6 | 1 |
| | 6 | 35 | | 422 | 37 | 43.3 | | 6.6 | 1 |
| | 6 | 33.2 | | 340 | 32 | 44.8 | | 5.6 | 1 |
| (Rodriguez et al., 1999; Denys et al., 2012; Bradham et al., 2013; Bradham et al., 2015) | 7 | 15.3 | | 244 | 42.58 | 24.04 | 1690 | 7.5 | 1 |
| | 7 | 13.9 | | 173 | 60.65 | 19.97 | 1400 | 6.4 | 1 |
| | 7 | 14.5 | | 6900 | 139.41 | 15.97 | 1090 | 5.5 | 1 |
| | 7 | 39.5 | | 280 | 77.88 | 4.482 | 62 | 2.3 | 1 |
| | 7 | 14.3 | | 4490 | 140.13 | 14.4 | 948 | 2.7 | 1 |
| | 7 | 17 | | 491 | 42.83 | 12.95 | 1800 | 6.9 | 1 |
| | 7 | 18.6 | | 207 | 39.38 | 21.52 | 2320 | 6.7 | 1 |
| | 7 | 26.4 | | 182 | 22.94 | 21.93 | 780 | 6.9 | 1 |
| | 7 | 48.2 | | 990 | 17.65 | 12.52 | 1060 | 6.4 | 1 |
| | 7 | 49.2 | | 829 | 15.67 | 10.22 | 631 | 6.5 | 1 |
| | 7 | 51.1 | | 379 | 14.38 | 10.66 | 178 | 5.1 | 1 |
| | 7 | 11.4 | | 837 | 276.28 | 14.74 | 1310 | 7.1 | 1 |
| | 7 | 42.3 | | 601 | 31.25 | 19.09 | 1020 | 5 | 1 |
| | 7 | 41.5 | | 1510 | 38.59 | 10.86 | 989 | 4 | 1 |
| | 7 | 16.2 | | 879 | 36.05 | 4.136 | 6740 | 5.9 | 1 |
| | 7 | 26.1 | | 322 | 26.56 | 37.69 | 1170 | 6.2 | 1 |
| | 7 | 34.9 | | 462 | 46.65 | 66.85 | 1410 | 6.2 | 1 |
| | 7 | 20.7 | | 401 | 40.96 | 53.48 | 1440 | 5.6 | 1 |
| | 7 | 34.7 | | 422 | 35.21 | 47 | 1770 | 5.9 | 1 |
| | 7 | 32.8 | | 340 | 23.17 | 20.93 | 1640 | 5.7 | 1 |
| | 7 | 46 | | 396 | 20.35 | 12.75 | 1280 | 5.6 | 1 |
| | 7 | 28.7 | | 197 | 23.44 | 27.36 | 50 | 5.2 | 1 |
| | 7 | 22.9 | | 884 | 29.96 | 28.44 | 51 | 6.4 | 1 |
| | 7 | 17.8 | | 293 | 35.51 | 40.12 | 34 | 6.5 | 1 |
| | 7 | 19.6 | | 223 | 28.33 | 34.15 | 45 | 5.4 | 1 |
| | 7 | 17.8 | | 494 | 37.84 | 28.01 | 36 | 6 | 1 |

| | | | | | | | | | |
|---|---|---|---|---|---|---|---|---|---|
| | 7 | 11.1 | | 738 | 31.19 | 19.74 | 43 | 6.6 | 1 |
| | 7 | 4.3 | | 777 | 174.23 | 2.74 | 194 | 2.9 | 1 |
| | 7 | 2.9 | | 943 | 106.44 | 0.678 | 46 | 3.1 | 1 |
| | 7 | 1.9 | | 898 | 199.07 | 1.7 | 159 | 3.3 | 1 |
| | 7 | 3.5 | | 668 | 179 | 3.68 | 184 | 3.5 | 1 |
| | 7 | 35.9 | | 981 | 31.95 | 11.35 | 861 | 8.3 | 1 |
| | 7 | 44.6 | | 246 | 20.48 | 22.98 | 197 | 8.8 | 1 |
| | 7 | 23.5 | | 108 | 33.66 | 26.45 | 157 | 7.8 | 1 |
| | 7 | 22.8 | | 184 | 31.24 | 20.71 | 16 | 6.4 | 1 |
| | 7 | 21.5 | | 965 | 97.66 | 72.08 | 24 | 5.7 | 1 |
| | 7 | 6.4 | | 573 | 30.35 | 9.06 | 66 | 6.6 | 1 |
| | 7 | 14 | | 583 | 25.49 | 4.7 | 25 | 7.6 | 1 |
| | 7 | 20.2 | | 239 | 23.86 | 10.7 | 4 | 8.6 | 1 |
| | 7 | 28.8 | | 313 | 19.15 | 18.86 | 16 | 5.2 | 1 |
| (Rodriguez et al., 1999; Bradham et al., 2013; Bradham et al., 2015) | 8 | 38.1 | | 676 | | | | | 1 |
| | 8 | 52.4 | | 313 | | | | | 1 |
| | 8 | | | 1663 | | | | | 1 |
| | 8 | | | 15952 | | | | | 1 |
| | 8 | 32 | | 290 | | | | | 1 |
| | 8 | 34.4 | | 388 | | | | | 1 |
| | 8 | 43.35 | | 382 | | | | | 1 |
| | 8 | 38.9 | | 364 | | | | | 1 |
| | 8 | | | 149 | | | | | 1 |
| | 8 | 17.8 | | 234 | | | | | 1 |
| | 8 | 23.6 | | 367 | | | | | 1 |
| | 8 | 19 | | 300 | | | | | 1 |
| | 8 | 50.7 | | 181 | | | | | 1 |
| | 8 | 17 | | 1230 | | | | | 1 |
| | 8 | 18 | | 394 | | | | | 1 |
| | 8 | 5 | | 1492 | | | | | 1 |
| | 8 | | | 689 | | | | | 1 |
| | 8 | | | 490 | | | | | 1 |
| | 8 | | | 90 | | | | | 1 |
| | 8 | 100 | | 1239 | | | | | 1 |
| | 8 | | | 1414 | | | | | 1 |
| | 8 | | | 1767 | | | | | 1 |
| | 8 | | | 2961 | | | | | 1 |
| | 8 | | | 191 | | | | | 1 |
| | 8 | 31 | | 150 | | | | | 1 |
| | 8 | 7 | | 268 | | | | | 1 |
| | 8 | 5 | | 724 | | | | | 1 |
| | 8 | 60.2 | | 200 | | | | | 1 |
| | 8 | 18.6 | | 3957 | | | | | 1 |
| | 8 | | | 591 | | | | | 1 |
| | 8 | 13 | | 647 | | | | | 1 |
| | 8 | | | 41051 | | | | | 1 |
| | 8 | 44.1 | | 590 | | | | | 1 |
| | 8 | 41.8 | | 1400 | | | | | 1 |
| | 8 | | | 105 | | | | | 1 |
| | 8 | 15 | | 123 | | | | | 1 |
| | 8 | 20 | | 1000 | | | | | 1 |
| | 8 | 19 | | 549 | | | | | 1 |
| | 8 | 28 | | 339 | | | | | 1 |
| | 8 | 93 | | 514 | | | | | 1 |
| | 8 | 40.3 | | 312 | | | | | 1 |
| | 8 | 42.2 | | 983 | | | | | 1 |
| | 8 | 36.7 | | 390 | | | | | 1 |
| | 8 | 23.8 | | 813 | | | | | 1 |
| | 8 | 21.2 | | 368 | | | | | 1 |
| | 8 | 23.5 | | 516 | | | | | 1 |
| | 8 | 24 | | 301 | | | | | 1 |
| | 8 | 13 | | 1412 | | | | | 1 |
| (Juhasz et al., 2007b; | 9 | 19.2 | 15 | 22 | 13 | 14.4 | 222 | 8.3 | 0 |
| | 9 | 33 | 38 | 319 | 17.3 | 15.8 | 232 | 8.8 | 0 |
| | 9 | 20.4 | 17 | 421 | 40.3 | 24 | 417 | 8.5 | 0 |

| | | | | | | | | | |
|---|---|---|---|---|---|---|---|---|---|
| Bradham et al., 2013; Bradham et al., 2015) | 9 | 27 | 28 | 208 | 27.3 | 19.2 | 520 | 8.3 | 0 |
| | 9 | 24.6 | 24 | 564 | 39.6 | 18.2 | 583 | 8.6 | 0 |
| | 9 | 16.8 | 11 | 246 | 34.1 | 39.4 | 296 | 6 | 0 |
| | 9 | 36.6 | 44 | 129 | 4.8 | 6.2 | 280 | 6.6 | 0 |
| | 9 | 13.8 | 6 | 463 | 23.8 | 17 | 470 | 5.9 | 0 |
| | 9 | 26.4 | 27 | 227 | 20.6 | 15.3 | 482 | 7.6 | 0 |
| | 9 | 25.2 | 25 | 198 | 19.5 | 10.6 | 521 | 8.6 | 0 |
| | 9 | 33.6 | 39 | 147 | 11.8 | 12.3 | 231 | 9 | 0 |
| | 9 | 39 | 48 | 1345 | 24.2 | 8.4 | 1410 | 8.7 | 0 |
| | 9 | 17.4 | 12 | 3601 | 117.2 | 93.3 | 3795 | 6 | 0 |
| | 9 | 17.4 | 12 | 1834 | 62 | 75.1 | 3048 | 5.9 | 0 |
| | 9 | 34.8 | 41 | 1346 | 53.5 | 20.8 | 2609 | 6 | 0 |
| | 9 | 24 | 23 | 39 | 8.7 | 9.9 | 2204 | 5.4 | 0 |
| | 9 | 21 | 18 | 1458 | 39.4 | 29.8 | 4358 | 5 | 0 |
| | 9 | 18 | 13 | 482 | 24.8 | 16.9 | 2515 | 4.6 | 0 |
| | 9 | 19.2 | 15 | 1465 | 93.9 | 98.9 | 4962 | 5.3 | 0 |
| | 9 | 24 | 23 | 83 | 7.1 | 9.1 | 548 | 5.3 | 0 |
| | 9 | 15.6 | 9 | 315 | 83.1 | 79 | 6801 | 4.7 | 0 |
| | 9 | 32.4 | 37 | 71 | 5.5 | 3.8 | 708 | 7.1 | 0 |
| | 9 | 37.8 | 46 | 3034 | 43 | 24.2 | 8159 | 6.8 | 0 |
| | 9 | 31.2 | 35 | 12781 | 254.1 | 12.9 | 699 | 8.7 | 0 |
| | 9 | 25.2 | 25 | 7099 | 189.2 | 23.5 | 786 | 6.1 | 0 |
| | 9 | 31.8 | 36 | 11280 | 245 | 12.8 | 658 | 9 | 0 |
| | 9 | 24.6 | 24 | 11013 | 263.6 | 16.2 | 795 | 6.6 | 0 |
| | 9 | 21 | 18 | 1063 | 23.7 | 9.9 | 335 | 8.5 | 0 |
| | 9 | 16.2 | 10 | 606 | 24.1 | 11.2 | 614 | 8.1 | 0 |
| | 9 | 10.8 | 1 | 422 | 80.8 | 38.6 | 781 | 6 | 0 |
| | 9 | 11.16 | 1.6 | 142 | 42.7 | 36 | 508 | 6.3 | 0 |
| | 9 | 10.92 | 1.2 | 358 | 40.6 | 24.1 | 466 | 5.4 | 0 |
| | 9 | 10.86 | 1.1 | 375 | 73.1 | 37.6 | 768 | 6 | 0 |
| | 9 | 15.6 | 9 | 50 | 18 | 8.3 | 230 | 8.2 | 0 |
| | 9 | 15.6 | 9 | 34 | 19 | 11 | 290 | 8.2 | 0 |
| | 9 | 12.9 | 4.5 | 41 | 20 | 7.8 | 70 | 8.2 | 0 |
| | 9 | 14.4 | 7 | 13 | 10 | 5.9 | 180 | 8.1 | 0 |
| | 9 | 13.2 | 5 | 61 | 18 | 6.1 | 120 | 7.9 | 0 |
| (Juhasz et al., 2007b; Brattin and Casteel, 2013) | 10 | 40 | | 312 | | | | | 1 |
| | 10 | 42 | | 983 | | | | | 1 |
| | 10 | 37 | | 390 | | | | | 1 |
| | 10 | 24 | | 813 | | | | | 1 |
| | 10 | 21 | | 368 | | | | | 1 |
| | 10 | 24 | | 516 | | | | | 1 |
| | 10 | 18 | | 234 | | | | | 1 |
| | 10 | 24 | | 367 | | | | | 1 |
| | 10 | 38 | | 676 | | | | | 1 |
| | 10 | 52 | | 313 | | | | | 1 |
| | 10 | 44 | | 74 | | | | | 1 |
| | 10 | 37 | | 73 | | | | | 1 |
| | 10 | 47 | | 320 | | | | | 1 |
| | 10 | 26 | | 3500 | | | | | 1 |
| (Juhasz et al., 2007a, b) | 11 | 72.2 | | 267 | 17.6 | 22.2 | 234 | 8.8 | 1 |
| | 11 | 41.6 | | 42 | 13.7 | 18.3 | 385 | 8.4 | 1 |
| | 11 | 20 | | 1114 | 68.3 | 16.3 | 874 | 7.8 | 1 |
| | 11 | 10.1 | | 257 | 25.8 | 27.8 | 242 | 6.4 | 1 |
| | 11 | 22.5 | | 751 | 14.5 | 10.8 | 422 | 8.3 | 1 |
| | 11 | 80.5 | | 91 | 10 | 5.1 | 130 | 7.5 | 1 |
| | 11 | 29.3 | | 713 | 98.6 | 94.7 | 3144 | 5.7 | 1 |
| | 11 | 43.8 | | 228 | 17.9 | 22.4 | 2941 | 5.2 | 1 |
| | 11 | 41.7 | | 807 | 23.5 | 10.9 | 546 | 7.6 | 1 |
| | 11 | 7 | | 577 | 24.6 | 17.6 | 468 | 6.6 | 1 |
| | 11 | 16.4 | | 190 | 21 | 8.3 | 200 | 8.6 | 1 |
| | 11 | 12.1 | | 88 | 21 | 9.6 | 370 | 8.1 | 1 |
| (Juhasz et al., 2007b; | 12 | 48.7 | | 990 | 20.9 | 11.8 | | 6.1 | 1 |
| | 12 | 49.7 | | 829 | 20.5 | 9.4 | | 6.3 | 1 |
| | 12 | 51.6 | | 379 | 18.9 | 9 | | 5 | 1 |
| | 12 | 11.2 | | 837 | 294.4 | 13.2 | | 7.2 | 1 |

| | | | | | | | | | |
|---|---|---|---|---|---|---|---|---|---|
| Juhasz et al., 2014) | 12 | 15.5 | | 244 | 46 | 21.7 | | 7.3 | 1 |
| | 12 | 14.1 | | 173 | 63.4 | 20.9 | | 6.6 | 1 |
| | 12 | 14.7 | | 6899 | 144.5 | 15 | | 5.2 | 1 |
| | 12 | 39.9 | | 280 | 72.3 | 3.9 | | 2.1 | 1 |
| | 12 | 14.5 | | 4495 | 120.1 | 12.3 | | 2.6 | 1 |
| | 12 | 42.1 | | 1513 | 34 | 10 | | 4 | 1 |
| (Oomen et al., 2002) | 13 | 51.39 | | 72 | | | | | 0 |
| | 13 | 12.78 | | 213 | | | | | 0 |
| | 13 | 60.3 | | 81 | | | | | 0 |
| (Roberts et al., 2006) | 14 | 13 | | 650 | | | | | 1 |
| | 14 | 13 | | 1412 | | | | | 1 |
| | 14 | 31 | | 189 | | | | | 1 |
| | 14 | 19 | | 300 | | | | | 1 |
| | 14 | 24 | | 301 | | | | | 1 |
| | 14 | 15 | | 125 | | | | | 1 |
| | 14 | 18 | | 394 | | | | | 1 |
| | 14 | 17 | | 1230 | | | | | 1 |
| | 14 | 5 | | 1492 | | | | | 1 |
| | 14 | 7 | | 268 | | | | | 1 |
| | 14 | 19 | | 339 | | | | | 1 |
| | 14 | 28 | | 546 | | | | | 1 |
| | 14 | 20 | | 1000 | | | | | 1 |
| | 14 | 5 | | 724 | | | | | 1 |
| (Ruby et al., 1996) | 15 | 48 | | 3900 | | | | 6.6 | 1 |
| | 15 | 20 | | 410 | | | | 7.8 | 1 |
| | 15 | 28 | | 170 | | | | 7.6 | 1 |
| (Wragg et al., 2011) | 16 | 8.62 | | 11300 | | | | | 1 |
| | 16 | 4.07 | | 17500 | | | | | 1 |
| | 16 | 7.88 | | 13500 | | | | | 1 |
| | 16 | 22.8 | | 11500 | | | | | 1 |
| | 16 | 38.7 | | 405 | | | | | 1 |
| | 16 | 43 | | 450 | | | | | 1 |
| | 16 | 39.1 | | 1180 | | | | | 1 |
| | 16 | 32.9 | | 5020 | | | | | 1 |
| | 16 | 21.9 | | 4650 | | | | | 1 |
| | 16 | 37 | | 676 | | | | | 1 |
| | 16 | 51 | | 313 | | | | | 1 |
| (Zhu et al., 2016) | 17 | 10.896 | | 8.95 | | | | 8.27 | 0 |
| | 17 | 10.956 | | 18.12 | | | | 8.01 | 0 |
| (Ollson et al., 2016) | 18 | 20.7 | | 170 | 13.6 | | | 6 | 0 |
| | 18 | 65.943 | | 2270 | 27.2 | | | 8.1 | 0 |
| | 18 | 55.251 | | 17400 | 66.4 | | | 7.8 | 0 |
| | 18 | 43.866 | | 9020 | 101 | | | 6.1 | 0 |
| | 18 | 71.982 | | 1780 | 27.9 | | | 7.8 | 0 |
| | 18 | 73.962 | | 1170 | 27 | | | 8.1 | 0 |
| (Xia et al., 2016) | 19 | 80.99 | | 333.67 | | | | 7.12 | 0 |
| | 19 | 42.38 | | 220.5 | | | | 5.68 | 0 |
| | 19 | 64.9025 | | 419.5 | | | | 4.45 | 0 |
| | 19 | 47.33 | | 714 | | | | 4.92 | 0 |
| | 19 | 86.93 | | 271 | | | | 7.66 | 0 |
| | 19 | 87.92 | | 166.5 | | | | 7.73 | 0 |
| | 19 | 80.99 | | 174.5 | | | | 7.31 | 0 |
| (Li et al., 2015) | 20 | 52.8 | | 36 | 30.4 | | | | 1 |
| | 20 | 16.2 | | 41 | 37.5 | | | | 1 |
| | 20 | 65 | | 119 | 26.5 | | | | 1 |
| | 20 | 27.2 | | 171 | 28.8 | | | | 1 |
| | 20 | 13.9 | | 75 | 69.5 | | | | 1 |
| | 20 | 6.38 | | 743 | 115 | | | | 1 |
| | 20 | 7.29 | | 1470 | 143 | | | | 1 |
| | 20 | 73.1 | | 22 | 30.7 | | | | 1 |
| | 20 | 34.1 | | 87 | 22.7 | | | | 1 |
| | 20 | 8.31 | | 861 | 41 | | | | 1 |
| | 20 | 14 | | 2556 | 219 | | | | 1 |
| | 20 | 30.1 | | 4172 | 18.2 | | | | 1 |
| | 21 | 21.36 | | 74.5 | 34.1 | 50 | | 7.4 | 0 |

| | | | | | | | | | |
|---|---|---|---|---|---|---|---|---|---|
| (Yin et al., 2015) | 21 | 20.34 | | 80 | 32.2 | 47.3 | | 7.2 | 0 |
| | 21 | 20.52 | | 78.3 | 33.2 | 48.6 | | 8.2 | 0 |
| | 21 | 14.76 | | 88.1 | 33.5 | 67.4 | | 5 | 0 |
| | 21 | 90.66 | | 843 | 51.2 | 56.1 | | 7.6 | 0 |
| | 21 | 18.36 | | 174.3 | 35.9 | 63.9 | | 5.3 | 0 |
| | 21 | 14.58 | | 38.1 | 39.7 | 83.6 | | 6.1 | 0 |
| | 21 | 23.28 | | 143.3 | 27.5 | 53.8 | | 7.6 | 0 |
| | 21 | 13.5 | | 33.9 | 22.3 | 46.3 | | 7.9 | 0 |
| | 21 | 14.82 | | 32.5 | 22.4 | 46.3 | | 7.7 | 0 |
| | 21 | 41.58 | | 419.9 | 25.9 | 38.7 | | 7.3 | 0 |
| | 21 | 15.72 | | 15.5 | 22.3 | 52.2 | | 5.6 | 0 |
| | 21 | 70.86 | | 287.3 | 36.8 | 47.2 | | 7.3 | 0 |
| | 21 | 16.26 | | 146.3 | 33.6 | 69 | | 4.7 | 0 |
| | 21 | 22.8 | | 58.8 | 27.5 | 58.8 | | 7.6 | 0 |
| | 21 | 18.18 | | 95.2 | 32.6 | 61.3 | | 7.2 | 0 |
| | 21 | 13.32 | | 24.2 | 34.1 | 74.9 | | 7.5 | 0 |
| | 21 | 16.8 | | 160.7 | 35.5 | 71.5 | | 5.8 | 0 |
| (Wilson et al., 2014) | 22 | 12.244 | | 22.2 | | | 152 | 4.29 | 0 |
| | 22 | 13.5992 | | 12.1 | | | 80.8 | 5.99 | 0 |
| | 22 | 11.2528 | | 8.3 | | | 40 | 6.25 | 0 |
| (Das et al., 2013) | 23 | 13.62 | | 7 | 46.179 | | | 6.7 | 0 |
| | 23 | 22.98 | | 16 | 42.372 | | | 6.8 | 0 |
| | 23 | 26.76 | | 17 | 26.632 | | | 7.1 | 0 |
| | 23 | 27.9 | | 19 | 27.378 | | | 6.8 | 0 |
| | 23 | 24.6 | | 48 | 37.902 | | | 6.8 | 0 |
| | 23 | 31.32 | | 108 | 24.918 | | | 7.1 | 0 |
| | 23 | 30.84 | | 183 | 28.923 | | | 7.2 | 0 |
| | 23 | 37.98 | | 417 | 39.487 | | | 7.3 | 0 |
| (Appleton et al., 2012) | 24 | 22.58 | | 32 | | | | | 0 |
| | 24 | 30.5 | | 25 | | | | | 0 |
| | 24 | 25.55 | | 313 | | | | | 0 |
| | 24 | 9.71 | | 36 | | | | | 0 |
| | 24 | 6.74 | | 120 | | | | | 0 |
| | 24 | 18.62 | | 26 | | | | | 0 |
| | 24 | 23.57 | | 47 | | | | | 0 |
| | 24 | 12.68 | | 1009 | | | | | 0 |
| | 24 | 9.71 | | 94 | | | | | 0 |
| | 24 | 15.65 | | 42 | | | | | 0 |
| | 24 | 5.75 | | 72 | | | | | 0 |
| (Cave et al., 2013) | 25 | 3.374 | | 31.9 | | | | | 0 |
| | 25 | 3.275 | | 23.1 | | | | | 0 |
| | 25 | 4.067 | | 51 | | | | | 0 |
| | 25 | 4.067 | | 27.9 | | | | | 0 |
| | 25 | 3.176 | | 24.3 | | | | | 0 |
| | 25 | 11.69 | | 55 | | | | | 0 |
| | 25 | 3.077 | | 23.3 | | | | | 0 |
| | 25 | 2.978 | | 43.8 | | | | | 0 |
| | 25 | 4.463 | | 54.8 | | | | | 0 |
| | 25 | 3.473 | | 41.8 | | | | | 0 |
| | 25 | 4.562 | | 39.3 | | | | | 0 |
| | 25 | 5.75 | | 44.3 | | | | | 0 |
| | 25 | 3.671 | | 23.3 | | | | | 0 |
| | 25 | 4.166 | | 28.9 | | | | | 0 |
| | 25 | 3.671 | | 52.2 | | | | | 0 |
| | 25 | 4.859 | | 60 | | | | | 0 |
| | 25 | 5.453 | | 57.9 | | | | | 0 |
| | 25 | 3.077 | | 33.1 | | | | | 0 |
| | 25 | 3.275 | | 18.8 | | | | | 0 |
| | 25 | 3.374 | | 43.8 | | | | | 0 |
| | 25 | 4.265 | | 44.3 | | | | | 0 |
| | 25 | 3.671 | | 42 | | | | | 0 |
| | 25 | 3.374 | | 35 | | | | | 0 |
| | 25 | 4.166 | | 27.4 | | | | | 0 |
| | 25 | 3.572 | | 50.1 | | | | | 0 |
| | 25 | 6.344 | | 43.8 | | | | | 0 |

| | | | | | | | | | |
|---|---|---|---|---|---|---|---|---|---|
| | 25 | 7.433 | | 51.5 | | | | | 0 |
| | 25 | 2.78 | | 33.8 | | | | | 0 |
| | 25 | 5.453 | | 61.7 | | | | | 0 |
| | 25 | 3.869 | | 31.3 | | | | | 0 |
| | 25 | 2.483 | | 20.9 | | | | | 0 |
| | 25 | 3.473 | | 21.3 | | | | | 0 |
| | 25 | 4.661 | | 35.4 | | | | | 0 |
| | 25 | 3.176 | | 38.6 | | | | | 0 |
| | 25 | 2.186 | | 19.4 | | | | | 0 |
| | 25 | 2.582 | | 19.3 | | | | | 0 |
| | 25 | 3.77 | | 24.4 | | | | | 0 |
| | 25 | 1.988 | | 17.4 | | | | | 0 |
| | 25 | 3.671 | | 30.4 | | | | | 0 |
| | 25 | 5.057 | | 43.8 | | | | | 0 |
| | 25 | 6.146 | | 61 | | | | | 0 |
| | 25 | 6.74 | | 70.4 | | | | | 0 |
| | 25 | 2.483 | | 19.6 | | | | | 0 |
| | 25 | 2.879 | | 23 | | | | | 0 |
| | 25 | 5.354 | | 50.8 | | | | | 0 |
| | 25 | 5.156 | | 32.3 | | | | | 0 |
| | 25 | 3.275 | | 24.9 | | | | | 0 |
| | 25 | 2.879 | | 25.8 | | | | | 0 |
| | 25 | 3.275 | | 18.5 | | | | | 0 |
| | 25 | 3.374 | | 32.7 | | | | | 0 |
| (Mingot et al., 2011) | 26 | 43.28571429 | | 7 | 7.4 | 7.9 | 520 | 7.96 | 0 |
| | 26 | 44.22439024 | | 8.2 | 8.5 | 8.8 | 550 | 8.3 | 0 |
| | 26 | 35.325 | | 6.4 | 12.1 | 11.4 | 2110 | 7.92 | 0 |
| | 26 | 32.4 | | 7 | 13.8 | 12.4 | 2540 | 8 | 0 |
| | 26 | 34.2 | | 5.7 | 13.7 | 11.4 | 600 | 8.41 | 0 |
| | 26 | 31.74098361 | | 6.1 | 13.5 | 11.5 | 610 | 8.55 | 0 |
| | 26 | 43.25454545 | | 5.5 | 10.4 | 9.6 | 670 | 8.29 | 0 |
| | 26 | 37.83934426 | | 6.1 | 9.7 | 6.7 | 640 | 8.82 | 0 |
| | 26 | 30.80869565 | | 11.5 | 9.8 | 8.3 | 590 | 8.68 | 0 |
| | 26 | 31.2 | | 8.4 | 8 | 6.8 | 380 | 8.96 | 0 |
| | 26 | 33.86666667 | | 9 | 11.4 | 6.4 | 530 | 8.92 | 0 |
| | 26 | 31.5 | | 10 | 12.3 | 6.4 | 580 | 8.66 | 0 |
| | 26 | 36.87567568 | | 7.4 | 11.8 | 7.6 | 550 | 8.83 | 0 |
| | 26 | 35.8056338 | | 7.1 | 10 | 6.1 | 450 | 8.9 | 0 |
| | 26 | 40.11891892 | | 7.4 | 9 | 7.7 | 580 | 8.76 | 0 |
| | 26 | 36.03050847 | | 5.9 | 8.5 | 6.8 | 600 | 8.38 | 0 |
| | 26 | 34.47692308 | | 6.5 | 12.1 | 8.4 | 510 | 8.78 | 0 |
| | 26 | 37.34754098 | | 6.1 | 11.9 | 9.2 | 570 | 9 | 0 |
| | 26 | 34.00952381 | | 6.3 | 7.1 | 6.4 | 520 | 8.83 | 0 |
| | 26 | 37.74545455 | | 4.4 | 7.3 | 7.6 | 570 | 8.62 | 0 |
| | 26 | 36.53898305 | | 5.9 | 9.7 | 7 | 670 | 8.89 | 0 |
| | 26 | 45.43404255 | | 4.7 | 6.6 | 6.7 | 490 | 8.4 | 0 |
| | 26 | 32.00487805 | | 8.2 | 10.1 | 6.9 | 450 | 8.33 | 0 |
| | 26 | 37.075 | | 4.8 | 8.4 | 5.8 | 380 | 8.45 | 0 |
| | 26 | 32.64444444 | | 5.4 | 9.2 | 8.2 | 530 | 7.99 | 0 |
| | 26 | 36.53333333 | | 5.4 | 10 | 8 | 530 | 8.46 | 0 |
| | 26 | 32.97966102 | | 5.9 | 8 | 6.1 | 500 | 8.56 | 0 |
| | 26 | 29.98125 | | 6.4 | 7 | 4.5 | 380 | 8.66 | 0 |
| | 26 | 37.3875 | | 6.4 | 12.8 | 12.5 | 540 | 8.59 | 0 |
| | 26 | 33.52258065 | | 6.2 | 12.8 | 13.1 | 730 | 8.62 | 0 |
| | 26 | 33.81038961 | | 7.7 | 12.2 | 9.9 | 880 | 8.34 | 0 |
| | 26 | 35.89411765 | | 8.5 | 14.3 | 11.4 | 990 | 8.38 | 0 |
| (Lu et al., 2011) | 27 | 12.705 | | 31.41428571 | 34.85714286 | | | 7.174285714 | 0 |
| | 27 | 20.27 | | 25.75 | 32.23333333 | | | 7.436666667 | 0 |
| | 27 | 12.5893 | | 26.1 | 33.38333333 | | | 6.281666667 | 0 |
| | 27 | 14.841 | | 17.33333333 | 25.68333333 | | | 7.343333333 | 0 |
| | 28 | 11.58 | | 88.3 | 49.5 | | | 6.4 | 0 |

| | | | | | | | | | |
|---|---|---|---|---|---|---|---|---|---|
| (Cornejo-Ponce and Acarapi-Cartes, 2011) | 28 | 11.46 | | 85.4 | 50.1 | | | 6.7 | 0 |
| | 28 | 10.98 | | 89.5 | 48.7 | | | 6.4 | 0 |
| | 28 | 12.84 | | 74.5 | 49.1 | | | 7.3 | 0 |
| | 28 | 13.14 | | 73.3 | 48.3 | | | 7 | 0 |
| | 28 | 11.64 | | 72.7 | 47.9 | | | 7 | 0 |
| | 28 | 11.16 | | 94.4 | 50.5 | | | 7 | 0 |
| | 28 | 11.46 | | 95.8 | 51.1 | | | 7.2 | 0 |
| | 28 | 12 | | 91.1 | 49.5 | | | 7.3 | 0 |
| | 28 | 12.12 | | 93.8 | 49.1 | | | 7.3 | 0 |
| (Meunier et al., 2010) | 29 | 11.46 | | 77000 | | | | | 0 |
| | 29 | 10.572 | | 310000 | | | | | 0 |
| | 29 | 11.94 | | 21000 | | | | | 0 |
| | 29 | 12.54 | | 15000 | | | | | 0 |
| | 29 | 10.5 | | 73000 | | | | | 0 |
| | 29 | 10.278 | | 210000 | | | | | 0 |
| | 29 | 11.64 | | 19000 | | | | | 0 |
| | 29 | 11.16 | | 39000 | | | | | 0 |
| | 29 | 10.92 | | 49000 | | | | | 0 |
| | 29 | 38.4 | | 7200 | | | | | 0 |
| | 29 | 11.46 | | 62000 | | | | | 0 |
| | 29 | 11.28 | | 24000 | | | | | 0 |
| | 29 | 16.8 | | 24000 | | | | | 0 |
| | 29 | 11.4 | | 21000 | | | | | 0 |
| | 29 | 11.7 | | 320 | | | | | 0 |
| | 29 | 24.6 | | 410 | | | | | 0 |
| | 29 | 23.4 | | 650 | | | | | 0 |
| | 29 | 19.8 | | 740 | | | | | 0 |
| | 29 | 12.54 | | 7200 | | | | | 0 |
| | 29 | 10.488 | | 6300 | | | | | 0 |
| | 29 | 28.8 | | 460 | | | | | 0 |
| | 29 | 17.4 | | 890 | | | | | 0 |
| | 29 | 22.2 | | 2300 | | | | | 0 |
| | 29 | 18.6 | | 6800 | | | | | 0 |
| | 29 | 19.8 | | 5300 | | | | | 0 |
| | 29 | 18 | | 9200 | | | | | 0 |
| | 29 | 16.8 | | 200 | | | | | 0 |
| | 29 | 12.72 | | 34000 | | | | | 0 |
| | 29 | 15.18 | | 19000 | | | | | 0 |
| (Subacz et al., 2007) | 30 | 41.58 | | 44.4 | 6.27 | | | 7.38 | 0 |
| | 30 | 25.44 | | 24.7 | 4.27 | | | 7.73 | 0 |
| | 30 | 24.96 | | 700 | 3.77 | | | 8.79 | 0 |
| | 30 | 42.48 | | 17.1 | 2.09 | | | 8.47 | 0 |
| | 30 | 27.18 | | 15.5 | 11.3 | | | 6.99 | 0 |
| | 30 | 24.3 | | 323 | 10.4 | | | 6.4 | 0 |
| | 30 | 21.48 | | 12.3 | 83.9 | | | 6.4 | 0 |
| | 30 | 26.94 | | 743 | 8.71 | | | 5.9 | 0 |
| | 30 | 53.7 | | 315 | 4.78 | | | 5.35 | 0 |
| (Pouschat and Zagury, 2006) | 31 | 28.013 | | | | | | 6.1 | 0 |
| | 31 | 26.678 | | | | | | 6.17 | 0 |
| | 31 | 23.563 | | | | | | 6.37 | 0 |
| | 31 | 55.158 | | | | | | 7.13 | 0 |
| | 31 | 42.253 | | | | | | 7.17 | 0 |
| | 31 | 61.744 | | | | | | 6.2 | 0 |
| | 31 | 41.185 | | | | | | 6.18 | 0 |
| | 31 | 43.143 | | | | | | 6.35 | 0 |
| | 31 | 46.347 | | | | | | 6.62 | 0 |
| | 31 | 28.636 | | | | | | 6.64 | 0 |
| | 31 | 25.61 | | | | | | 5.47 | 0 |
| | 31 | 26.055 | | | | | | 5.27 | 0 |
| (Datta and Sarkar, 2005) | 32 | 72.12 | | 15 | | | 208 | | 1 |
| | 32 | 63.24 | | 16.5 | | | 4875 | | 1 |
| | 32 | 63.14 | | 14 | | | 6812 | | 1 |
| | 32 | 52.39 | | 17 | | | 1688 | | 1 |

| | | | | | | | | | |
|---|---|---|---|---|---|---|---|---|---|
| (Bradham et al., 2011) | 1 | 30.1 | | 990 | 20.9 | 11.8 | 0 | 6.1 | 1 |

Note: *, index equals 1 and 0 represent the RBA data from *in vivo* and *in vitro* approaches (using equations from Table 1 to convert), respectively.

## Reference

Appleton, J., Cave, M., Wragg, J., 2012. Anthropogenic and geogenic impacts on arsenic bioaccessibility in UK topsoils. Sci Total Environ 435, 21-29.

Bradham, K.D., Diamond, G.L., Scheckel, K.G., Hughes, M.F., Casteel, S.W., Miller, B.W., Klotzbach, J.M., Thayer, W.C., Thomas, D.J., 2013. Mouse assay for determination of arsenic bioavailability in contaminated soils. Journal of Toxicology and Environmental Health, Part A 76, 815-826.

Bradham, K.D., Nelson, C., Juhasz, A.L., Smith, E., Scheckel, K., Obenour, D.R., Miller, B.W., Thomas, D.J., 2015. Independent data validation of an in vitro method for the prediction of the relative bioavailability of arsenic in contaminated soils. Environ Sci Technol 49, 6312-6318.

Bradham, K.D., Scheckel, K.G., Nelson, C.M., Seales, P.E., Lee, G.E., Hughes, M.F., Miller, B.W., Yeow, A., Gilmore, T., Serda, S.M., 2011. Relative bioavailability and bioaccessibility and speciation of arsenic in contaminated soils. Environ Health Persp 119, 1629.

Brattin, W., Casteel, S., 2013. Measurement of arsenic relative bioavailability in swine. Journal of Toxicology and Environmental Health, Part A 76, 449-457.

Cave, M.R., Wragg, J., Harrison, H., 2013. Measurement modelling and mapping of arsenic bioaccessibility in Northampton, United Kingdom. Journal of Environmental Science and Health, Part A 48, 629-640.

Cornejo-Ponce, L., Acarapi-Cartes, J., 2011. Fractionation and bioavailability of arsenic in agricultural soils: Solvent extraction tests and their relevance in risk assessment. Journal of Environmental Science and Health, Part A 46, 1247-1258.

Das, S., Jean, J.-S., Kar, S., 2013. Bioaccessibility and health risk assessment of arsenic in arsenic-enriched soils, Central India. Ecotox Environ Safe 92, 252-257.

Datta, R., Sarkar, D., 2005. Consideration of soil properties in assessment of human health risk from exposure to arsenic-enriched soils. Integrated environmental assessment and management 1, 55-59.

Denys, S., Caboche, J., Tack, K., Rychen, G., Wragg, J., Cave, M., Jondreville, C., Feidt, C., 2012. In vivo validation of the unified BARGE method to assess the bioaccessibility of arsenic, antimony, cadmium, and lead in soils. Environ Sci Technol 46, 6252-6260.

Juhasz, A.L., Herde, P., Herde, C., Boland, J., Smith, E., 2015. Predicting arsenic relative bioavailability using multiple in vitro assays: validation of in vivo–in vitro correlations. Environ Sci Technol 49, 11167-11175.

Juhasz, A.L., Smith, E., Nelson, C., Thomas, D.J., Bradham, K., 2014. Variability associated with As in vivo–in vitro correlations when using different bioaccessibility methodologies. Environ Sci Technol 48, 11646-11653.

Juhasz, A.L., Smith, E., Weber, J., Rees, M., Rofe, A., Kuchel, T., Sansom, L., Naidu, R., 2007a. Comparison of in vivo and in vitro methodologies for the assessment of arsenic bioavailability in contaminated soils. Chemosphere 69, 961-966.

Juhasz, A.L., Smith, E., Weber, J., Rees, M., Rofe, A., Kuchel, T., Sansom, L., Naidu, R., 2007b. In vitro assessment of arsenic bioaccessibility in contaminated (anthropogenic and geogenic) soils. Chemosphere 69, 69-78.

Juhasz, A.L., Weber, J., Smith, E., Naidu, R., Rees, M., Rofe, A., Kuchel, T., Sansom, L., 2009. Assessment of four commonly employed in vitro arsenic bioaccessibility assays for predicting in vivo relative arsenic bioavailability in contaminated soils. Environ Sci Technol 43, 9487-9494.

Li, J., Li, K., Cui, X.-Y., Basta, N.T., Li, L.-P., Li, H.-B., Ma, L., 2015. In vitro bioaccessibility and in vivo relative bioavailability in 12 contaminated soils: Method comparison and method development. Sci Total Environ 532, 812-820.

Lu, Y., Yin, W., Huang, L., Zhang, G., Zhao, Y., 2011. Assessment of bioaccessibility and exposure risk of arsenic and lead in urban soils of Guangzhou City, China. Environ Geochem Hlth 33, 93-102.

Meunier, L., Walker, S.R., Wragg, J., Parsons, M.B., Koch, I., Jamieson, H.E., Reimer, K.J., 2010. Effects of soil composition and mineralogy on the bioaccessibility of arsenic from tailings and soil in gold mine districts of Nova Scotia. Environ Sci Technol 44, 2667-2674.

Mingot, J., De Miguel, E., Chacón, E., 2011. Assessment of oral bioaccessibility of arsenic in playground soil in Madrid (Spain): a three-method comparison and implications for risk assessment. Chemosphere 84, 1386-1391.
Ollson, C.J., Smith, E., Scheckel, K.G., Betts, A.R., Juhasz, A.L., 2016. Assessment of arsenic speciation and bioaccessibility in mine-impacted materials. J Hazard Mater 313, 130-137.
Oomen, A.G., Hack, A., Minekus, M., Zeijdner, E., Cornelis, C., Schoeters, G., Verstraete, W., Van de Wiele, T., Wragg, J., Rompelberg, C.J., 2002. Comparison of five in vitro digestion models to study the bioaccessibility of soil contaminants. Environ Sci Technol 36, 3326-3334.
Pouschat, P., Zagury, G.J., 2006. In vitro gastrointestinal bioavailability of arsenic in soils collected near CCA-treated utility poles. Environ Sci Technol 40, 4317-4323.
Roberts, S.M., Munson, J.W., Lowney, Y.W., Ruby, M.V., 2006. Relative oral bioavailability of arsenic from contaminated soils measured in the cynomolgus monkey. Toxicol Sci 95, 281-288.
Rodriguez, R.R., Basta, N.T., Casteel, S.W., Pace, L.W., 1999. An in vitro gastrointestinal method to estimate bioavailable arsenic in contaminated soils and solid media. Environ Sci Technol 33, 642-649.
Ruby, M.V., Davis, A., Schoof, R., Eberle, S., Sellstone, C.M., 1996. Estimation of lead and arsenic bioavailability using a physiologically based extraction test. Environ Sci Technol 30, 422-430.
Subacz, J.L., Barnett, M.O., Jardine, P.M., Stewart, M.A., 2007. Decreasing arsenic bioaccessibility/bioavailability in soils with iron amendments. Journal of Environmental Science and Health Part A 42, 1317-1329.
Wilson, S.C., Tighe, M., Paterson, E., Ashley, P.M., 2014. Food crop accumulation and bioavailability assessment for antimony (Sb) compared with arsenic (As) in contaminated soils. Environ Sci Pollut R 21, 11671-11681.
Wragg, J., Cave, M., Basta, N., Brandon, E., Casteel, S., Denys, S., Gron, C., Oomen, A., Reimer, K., Tack, K., 2011. An inter-laboratory trial of the unified BARGE bioaccessibility method for arsenic, cadmium and lead in soil. Sci Total Environ 409, 4016-4030.
Xia, Q., Peng, C., Lamb, D., Mallavarapu, M., Naidu, R., Ng, J.C., 2016. Bioaccessibility of arsenic and cadmium assessed for in vitro bioaccessibility in spiked soils and their interaction during the Unified BARGE Method (UBM) extraction. Chemosphere 147, 444-450.
Yin, N., Cui, Y., Zhang, Z., Wang, Z., Cai, X., Wang, J., 2015. Bioaccessibility and dynamic dissolution of arsenic in contaminated soils from Hunan, China. Journal of soils and sediments 15, 584-593.
Zhu, X., Yang, F., Wei, C., Liang, T., 2016. Bioaccessibility of heavy metals in soils cannot be predicted by a single model in two adjacent areas. Environ Geochem Hlth 38, 233-241.
